# The Women's Maternity Unit for Russia

**Published:** 1916-01-29

**Authors:** L. E. Beaufort, Consuelo Marlborough, Olive Cairns, Maud Selborne, Isabel Somerset, Mary Murray, V. Wolkoff, Gertrude Kinnell, L. B. Aldrich Blake, Mary Scharlieb, Louise Vinocradoff


*To the Editor of* The Hospital.

### The Women's Maternity Unit for Russia.

*To the Editor of* The Hospital.

Sir,—Russia is labouring to alleviate the unspeakable sufferings of the millions of peasants who are refugees from the war zone. You have already reported that the National Union of Women's Suffrage Societies has initiated a movement to give an opportunity for the women of Great Britain to share in some small degree the efforts of our Russian sisters. The first unit will start for Petrograd immediately to take over the maternity hospital now being built for it by the Tatiana Committee. Two medical women, with matron and fully trained nursing staff, will take medical stores and comforts and clothes for the women, children, and infants, and they hope, besides the sixteen beds in the hospital, to be able to undertake a large out-patient department. The hospital will have the gracious protection of the Empress Alexandra and the active patronage of the Grand Duchess Kyril and the Lady Georgiana Buchanan, the British Ambassadress. May we, as patrons of the work in this country, plead earnestly for support from all those whose hearts have been wrung by the awful tales of misery among the peasants, who are called upon to suffer all that we have escaped by being further from the battle line.

Surely, when we think of our own griefs and anxieties, we should also remember that these people too have their nearest and dearest in equal peril, and yet have the added horror of seeing their homes and all their possessions destroyed and their little ones sick and dying around them. We do not necessarily speak as members of the society which is organising these units, but as British women feeling strongly the claim of suffering and glad to join in a work which promises in so practical a way to be of use.

Should not those who start forth be able to carry with them the assurance that at least there are funds enough to keep the hospital going for six months? The Tatiana Committee have granted 1,000 roubles a month towards upkeep. We must provide at least as much—namely, about £100 a month—besides the heavy initial cost of salaries, outfit, journeys, and medical stores. Donations should be sent to the Hon. Treasurer, Russian Unit, 14 Great Smith Street, Westminster.—(Signed) L. E. Beaufort, Consuelo Marlborough, Olive Cairns, Maud Selborne, Isabel Somerset, Mary Murray, V. Wolkoff, Gertrude Kinnell, L. B. Aldrich Blake, Mary Scharlieb, Louise Vinogradoff.

